# Supplementary material for: Locomotion modulates olfactory learning through proprioception in C. elegans
Source: Nat Commun. 2023 Jul 27;14:4534. doi: 10.1038/s41467-023-40286-x (PMC10374624; doi:10.1038/s41467-023-40286-x)
Supplement: Supplementary file 1 — Supplementary Information [file 41467_2023_40286_MOESM1_ESM.pdf]

Locomotion modulates olfactory learning through proprioception in *C. elegans*

Xu Zhan, Chao Chen, Longgang Niu, Xinran Du, Ying Lei, Rui Dan, Zhao-Wen Wang, and Ping Liu

Supplementary Information

Supplementary Figures 1-14 and Supplementary Table 1.

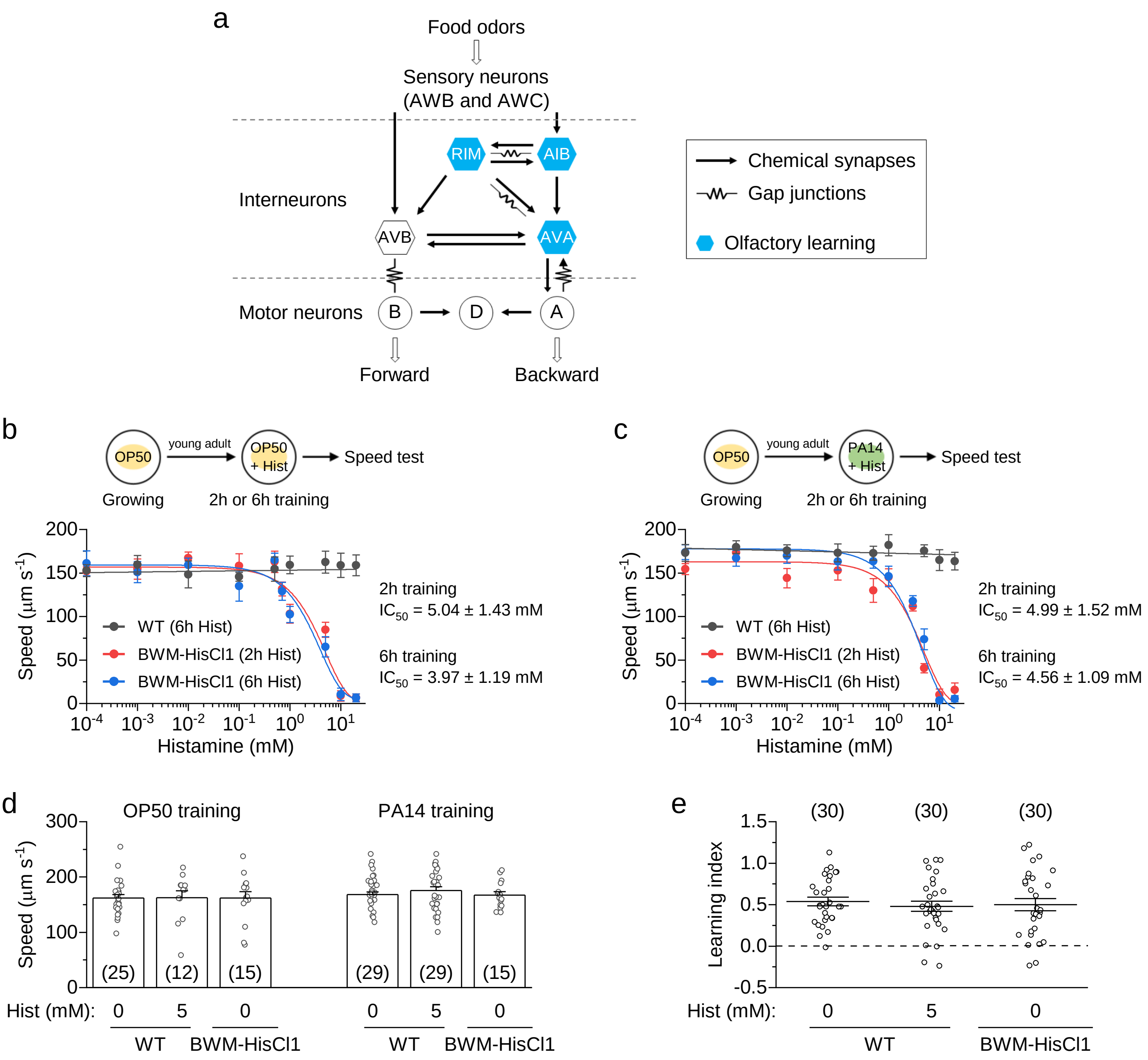

Supplementary Fig. 1. Histamine inhibits the locomotion speed of BWM-HisCl1 animals but does not affect either the locomotion speed or olfactory learning index of wild type. a Wiring diagram showing major synaptic connections of AVA, AIB, and RIM in the sensorimotor circuit. B-, D-, and A-type motor neurons are labeled as B, D, and A, respectively. **b, c** Histamine concentration-locomotion speed curves of wild type (WT) and BWM-HisCl1 animals trained with OP50 (**b**) or PA14 (**c**). IC_{50} represents the histamine concentration for half-maximal inhibition of the locomotion speed. Each data point represents results from 10-30 animals at one concentration of histamine. The solid lines are linear (black) and single exponential (red and blue) fits to the data. **d, e** Comparison of locomotion speed and olfactory learning index among WT control (zero histamine), WT treated with 5 mM histamine, and BWM-HisCl1 (zero histamine) following 6 hours of training with OP50 or PA14. No statistically significant difference among the three groups (one-way ANOVA with Tukey's post hoc test). Compared with WT control (zero histamine), p = 1, 1, 0.6872, 0.6992, and 0.9973 (**d**), 0.7806 and 0.8935 (**e**). Brackets contain the number of animals tested (**d**) or independent assays (**e**). Data are shown as means ± SEM. Source data are provided as a Source Data file.

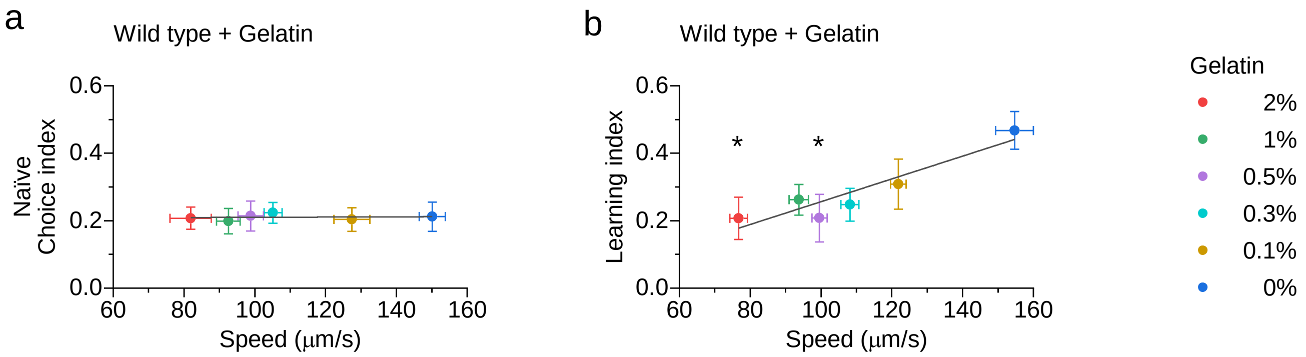

Supplementary Fig. 2. Slower locomotion speed in wild type impairs the olfactory learning index without altering the naïve choice index. Gelatin was added to assay plates at various concentrations to reduce locomotion speed. **a** Relationship of locomotion speed versus the naïve choice index. **b** Relationship of locomotion speed versus the olfactory learning index. Each data point represents results from 24-38 assays at one concentration of gelatin. In each assay, 5 and 100-150 animals were used for the speed test and choice test, respectively. Solid lines are linear fits to the data. * indicates $p < 0.05$ compared with 0% gelatin (one-way ANOVA with Tukey's post hoc test). p = 1, 0.9999, 1, 0.9999, and 1 (**a**), 0.0398, 0.1827, 0.0351, 0.1206, and 0.4696 (**b**). Data are shown as means ± SEM. Source data are provided as a Source Data file.

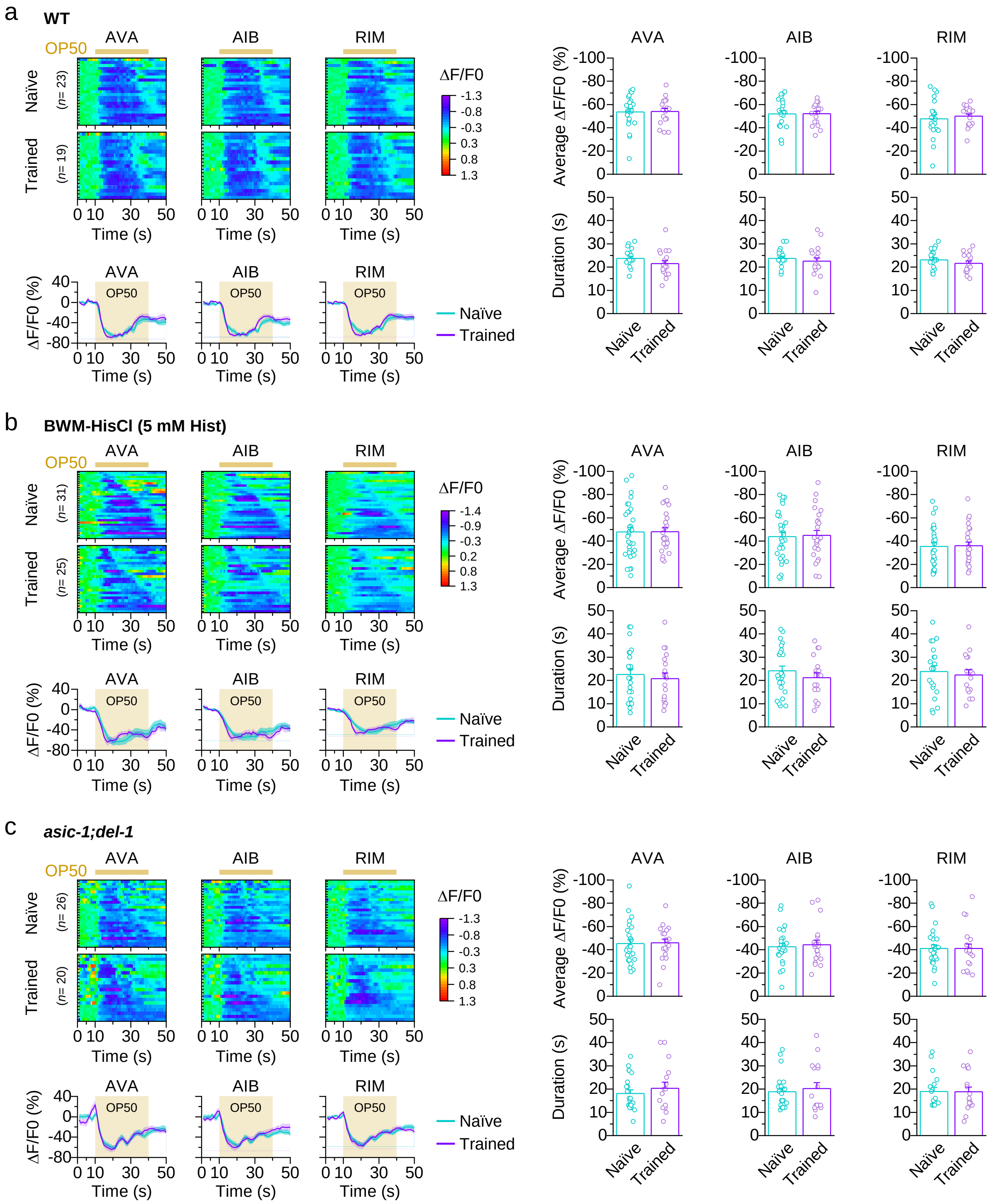

Supplementary Fig. 3. PA14 training does not affect OP50-induced Ca^{2+} drop in AVA, AIB, and RIM. Ca^{2+} signals were imaged in transgenic animals with cell-targeted expression of GCaMP6. Shown are heatmaps of Ca^{2+} signals (arranged by the response duration), the mean (solid line) and SEM (shaded area) of each group, and quantification of OP50-induced Ca^{2+} drop in naïve and trained wild type (**a**), BWM-HisCl1 animals with prior histamine treatment (**b**), and *asic-1(ok415);del-1(ok150)* double mutant (**c**). Each row of the heatmaps represents the Ca^{2+} signal of an individual animal. No

statistically significant difference between the naïve and trained groups in all cases (two-sided unpaired *t*-test). From left to right, p = 0.9186, 0.9584, and 0.5909 (top, **a**), 0.1705, 0.4841, and 0.3345 (bottom, **a**), 0.9811, 0.8605, and 0.7539 (top, **b**), 0.5987, 0.3423, and 0.66 (bottom, **b**), 0.9109, 0.7038, and 0.9855 (top, **c**), 0.4429, 0.6379, and 0.9835 (bottom, **c**). *n* represents the number of animals tested. Data are shown as mean ± SEM. Source data are provided as a Source Data file.

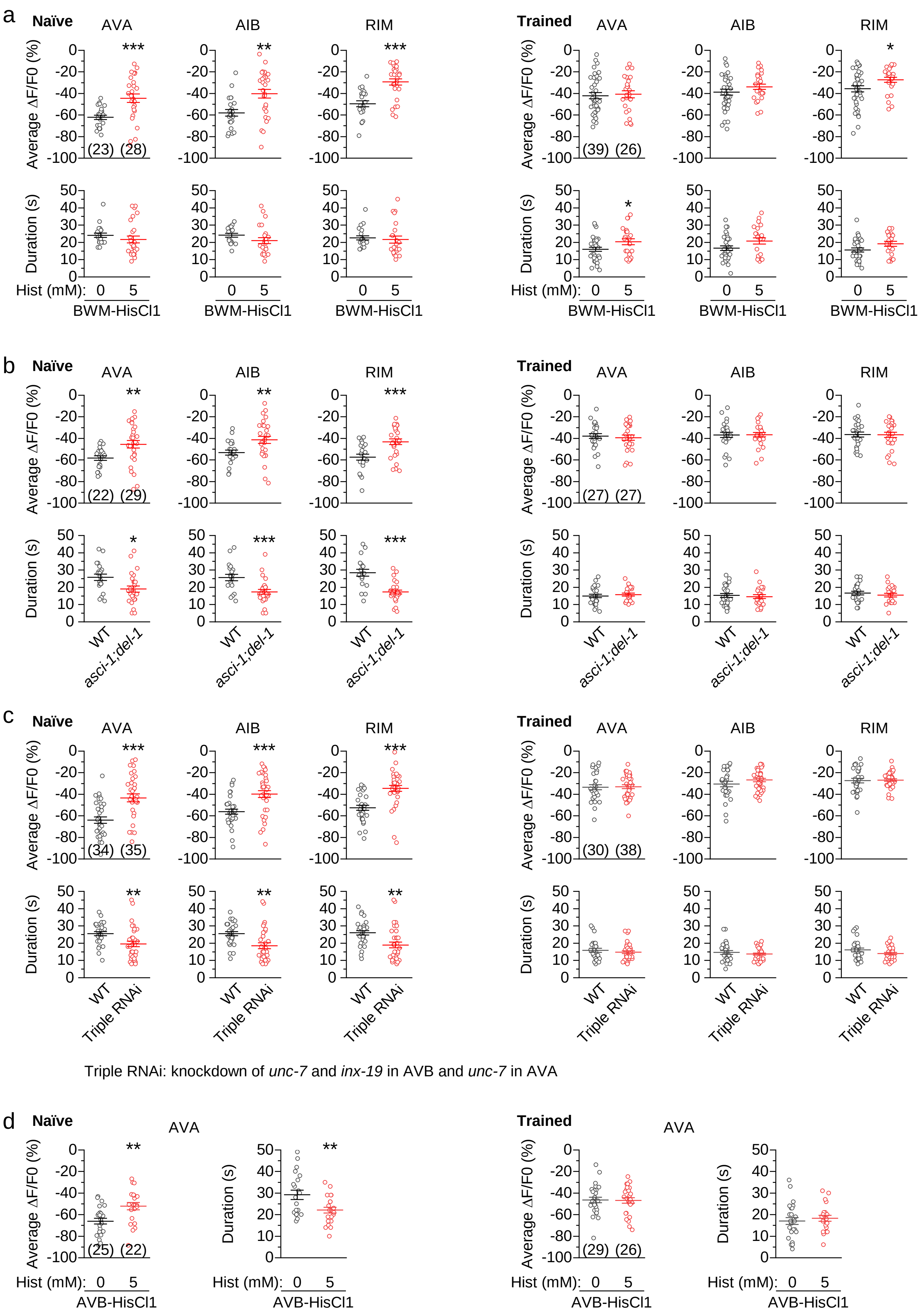
a
Naïve
Trained
AVA
AIB
RIM
Average ΔF/F0 (%)
Duration (s)
Hist (mM): 0 5
BWM-HisCl1
b
WT
asci-1;del-1
c
Triple RNAi
Triple RNAi: knockdown of *unc-7* and *inx-19* in AVB and *unc-7* in AVA
d
AVB-HisCl1

Supplementary Fig. 4. Locomotion inhibition and mechanoreceptor mutations reduce PA14-induced Ca^{2+} drops in AVA, AIB, and RIM in naïve but not trained animals. a Effects of locomotion inhibition in BWM-HisCl1 animals by histamine on PA14-induced Ca^{2+} drop. **b** Effects of *asic-1(ok415)* and *del-1(ok150)* mutations on PA14-induced Ca^{2+} drop. **c** Effects of triple RNAi (knockdown of *unc-7* and *inx-19* in AVB and *unc-7* in AVA) on PA14-induced Ca^{2+} drop. **d** Effects of acute inhibition of AVB in AVB-HisCl1 animals by histamine on PA14-induced Ca^{2+} drop. The data were from the same groups as those shown in Fig. 1c-f, Fig. 4c, Fig. 6a-d, and Fig. 7a, b. *, **, and *** indicate $p < 0.05$, $p < 0.01$, and $p < 0.001$, respectively, compared with either the zero histamine or wild type (WT) group (two-sided unpaired *t*-test). From left to right, p = 0.0005, 0.0014, < 0.0001, 0.7637, 0.2151, and 0.0374 (top, **a**), 0.3411, 0.1604, 0.695, 0.0355, 0.061, and 0.0593 (bottom, **a**), 0.0055, 0.0081, 0.0005, 0.6594, 0.9467, and 0.9465 (top, **b**), 0.0106, 0.0006, < 0.0001, 0.5658, 0.6686, and 0.4069 (bottom, **b**), < 0.0001, 0.0001, < 0.0001, 0.8562, 0.1896, and 0.8413 (top, **c**), 0.0046, 0.0016, 0.0018, 0.4174, 0.4328, and 0.0964 (bottom, **c**), 0.0017, 0.0072, 0.9001, and 0.5675 (**d**). Brackets contain the number of animals tested. Data are shown as mean ± SEM. Source data are provided as a Source Data file.

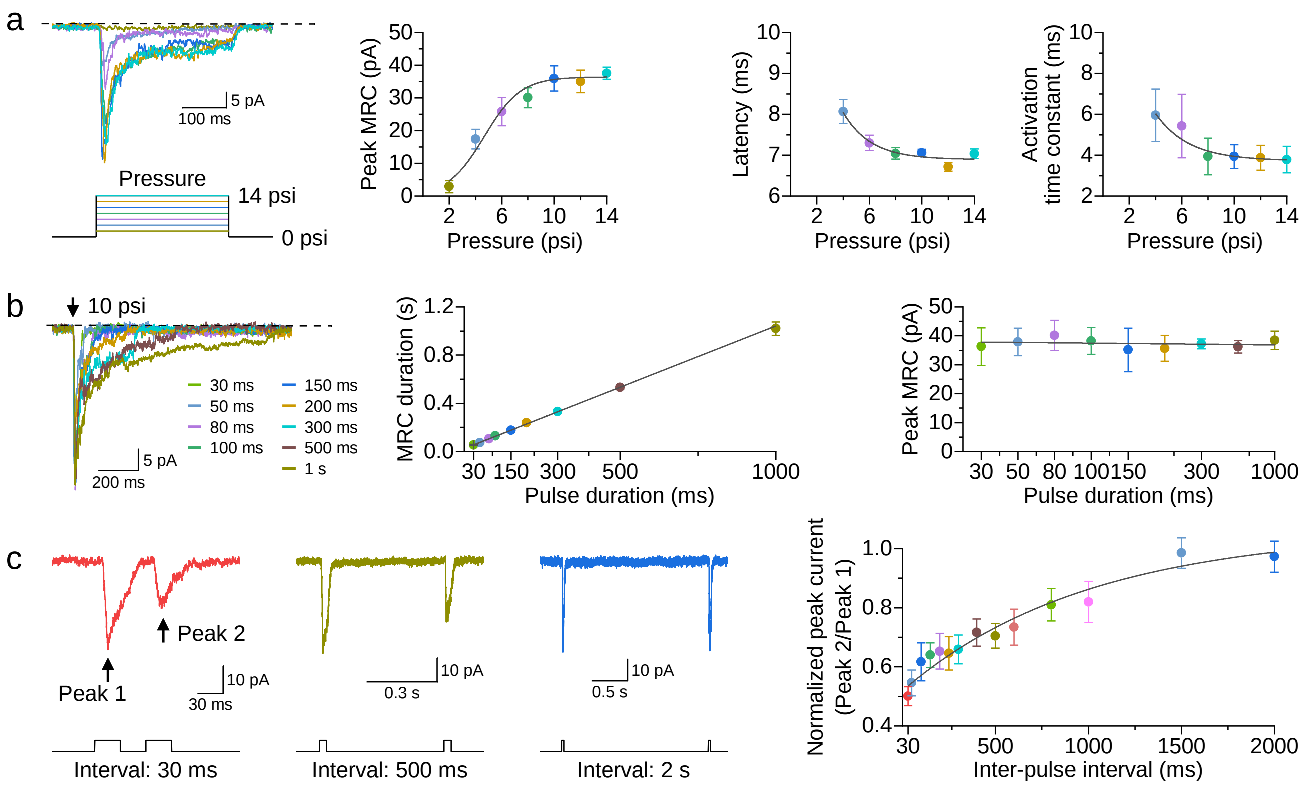

Supplementary Fig. 5. Biophysical properties of mechanoreceptor currents (MRCs) in VB6. a Effects of altering stimulation pressure on MRC peak current properties. The data of stimulation pressure versus peak MRC are fit to a Boltzmann function ($I=I_{max}/(1+\exp((X_{1/2}-X)/X_{slope}))$), whereas those of stimulation pressure versus MRC latency and activation rate are fit to a single exponential function. n = 6-7. **b** Effects of stimulation pulse (10 psi) duration (30-1,000 ms) on MRC duration and peak amplitude. Solid lines are linear fits to the data. n = 6-7. **c** MRCs in response to two-pulse stimuli (10 psi, 30 ms) at intervals ranging from 30 ms to 2 s. The solid line is a single exponential fit to the data. n = 6-9. n represents the number of cells recorded. Data are shown as means ± SEM. Source data are provided as a Source Data file.

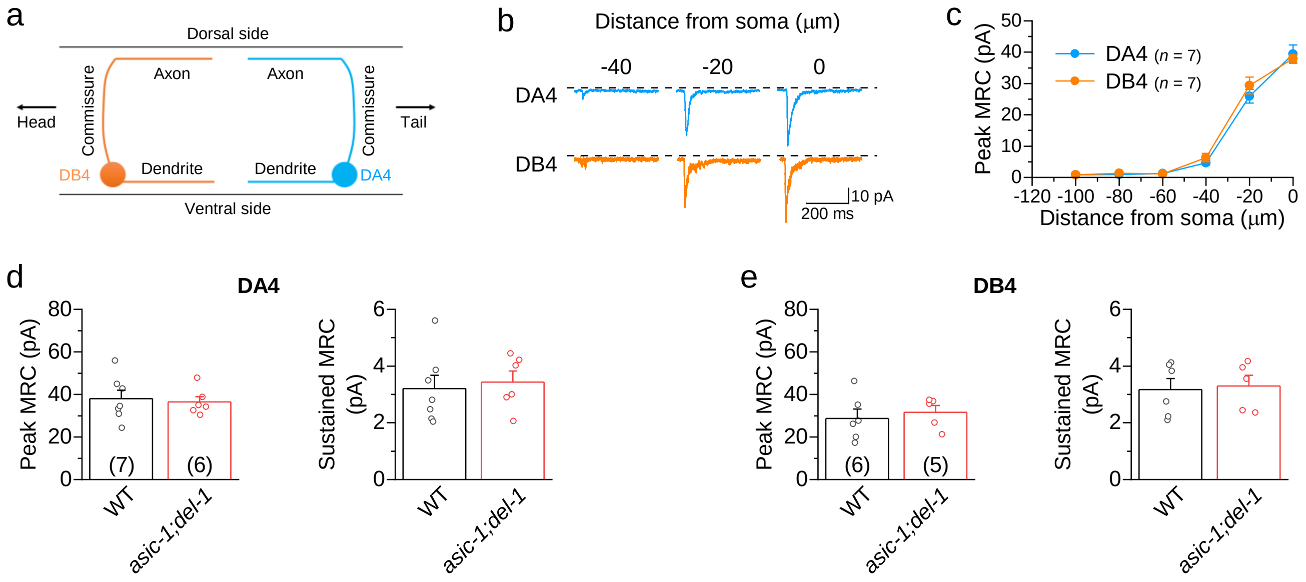

Supplementary Fig. 6. Mechanosensitivity of DA4 and DB4 does not depend on the functions of ASIC-1 and DEL-1. a Diagram showing the locations of somata, dendrites, and axons of DA4 and DB4. **b** Sample mechanoreceptor current (MRC) traces caused by pressure stimulation at different locations of DA4 and DB4. **c** Relationships of peak MRCs versus locations of stimuli. **d**, **e** Comparisons of peak and sustained MRCs of DA4 and DB4 between wild type (WT) and *asic-1(ok415);del-1(ok150)*. No statistically significant difference between WT and the mutant in all cases (two-sided unpaired *t*-test). From left to right, p = 0.7509 and 0.7082 (**d**), 0.6288 and 0.833 (**e**). Brackets contain the number of cells recorded (n). Data are shown as means ± SEM. Source data are provided as a Source Data file.

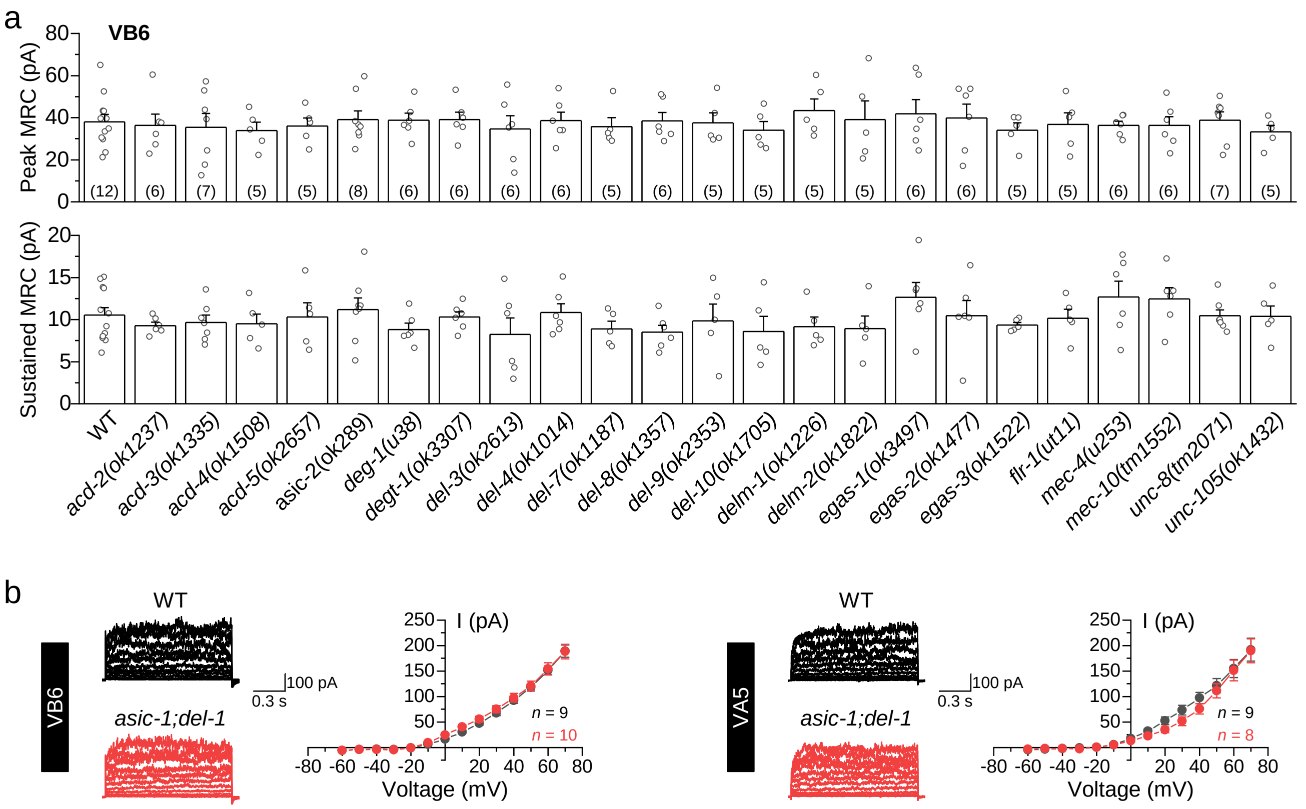

Supplementary Fig. 7. Mutations of other candidate DEG/ENaC/ASIC genes do not affect mechanoreceptor currents (MRCs) in VB6, and mutations of *asic-1* and *del-1* do not affect voltage-dependent currents in VB6 and VA5. a Comparisons of the peak MRC and the sustained MRC among wild type (WT) and mutants of candidate mechanosensitive channels. No significant difference between WT and the mutants (one-way ANOVA with Tukey's post hoc test). $p = 1$ for every comparison. **b** Whole-cell current of VB6 and VA5 caused by membrane voltage steps (-60 mV to +70 mV at 10-mV intervals) from a holding voltage of -60 mV. Shown are sample current traces and current-voltage relationships of WT and *asic-1(ok415);del-1(ok150)* mutant. Brackets contain the number of cells recorded (n). Data are shown as means ± SEM. Source data are provided as a Source Data file.

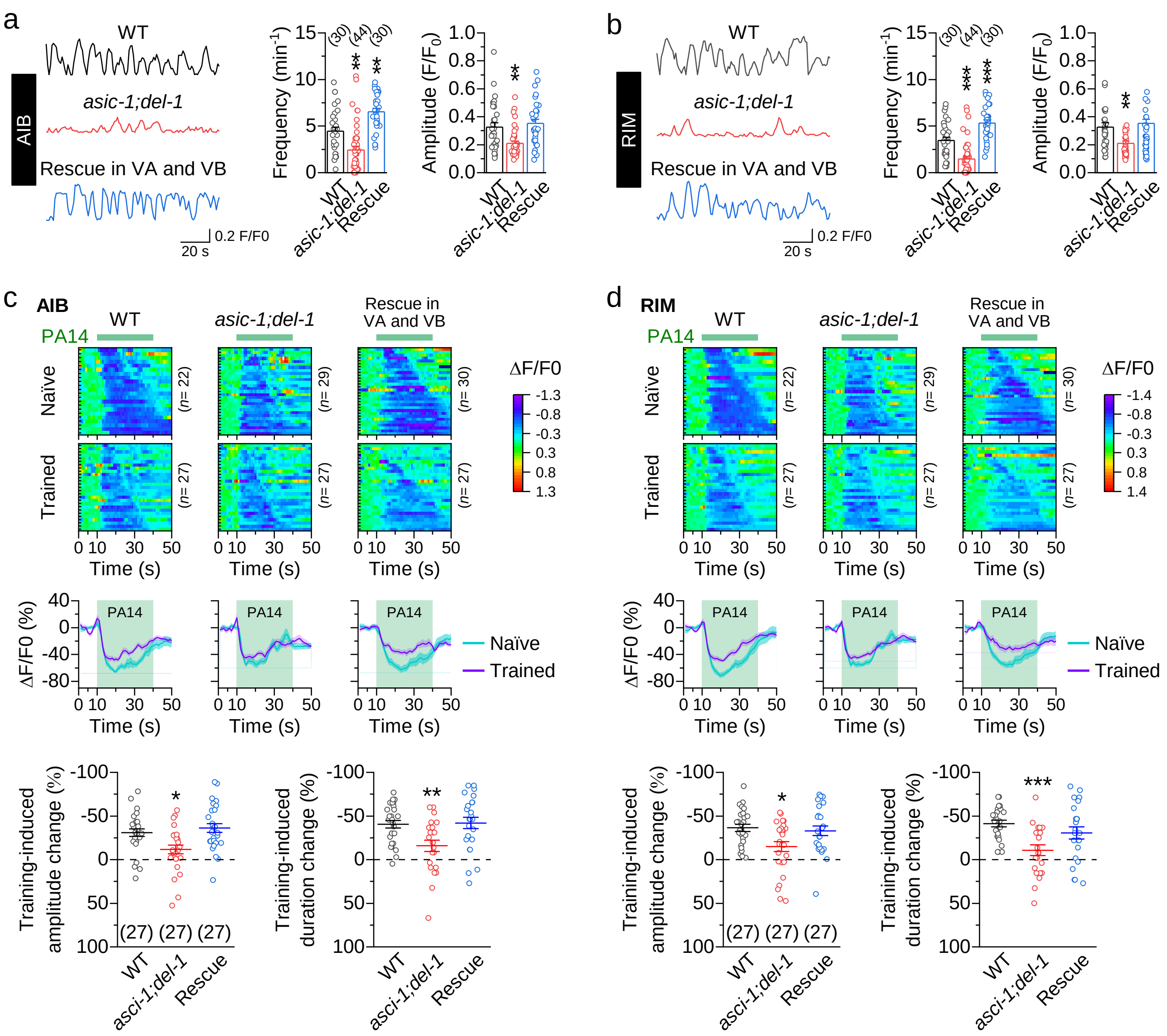

Supplementary Fig. 8. Mutations of *asic-1* and *del-1* inhibit AIB and RIM activity, and attenuate PA14-induced Ca^{2+} drop in AIB and RIM. a, b *asic-1(ok415);del-1(ok150)* mutant showed great decreases in the frequency and amplitude of Ca^{2+} transients in AIB and RIM compared with wild type (WT), which could be rescued by expressing wild-type *asic-1* and *del-1* in VA and VB using *Pdel-1*. **c, d** *asic-1(ok415);del-1(ok150)* mutant displayed less PA14 training-induced change of the Ca^{2+} drop in AIB and RIM than WT, and this phenotype could be rescued by expressing wild-type *asic-1* and *del-1* in VA and VB. Shown are the heatmaps of the Ca^{2+} signal of individual animals arranged by the response duration (top), the mean (solid line) and SEM (shaded area) of each group (middle), and statistical comparisons of the training-induced changes of the Ca^{2+} drop (bottom). *, **, and *** indicate $p < 0.05$, $p < 0.01$, and $p < 0.001$, respectively, compared with WT (one-way ANOVA with Tukey's post hoc test). From left to right, p = 0.0015, 0.0029, 0.0058, and 0.7664 (**a**), < 0.0001, 0.0005, 0.0014, and 0.5691 (**b**), 0.0172, 0.7212, 0.0092, and 0.9826 (**c**), 0.0101, 0.8907, 0.0007, and 0.364 (**d**). Brackets contain the number of animals tested (n). Data are shown as means ± SEM. Source data are provided as a Source Data file.

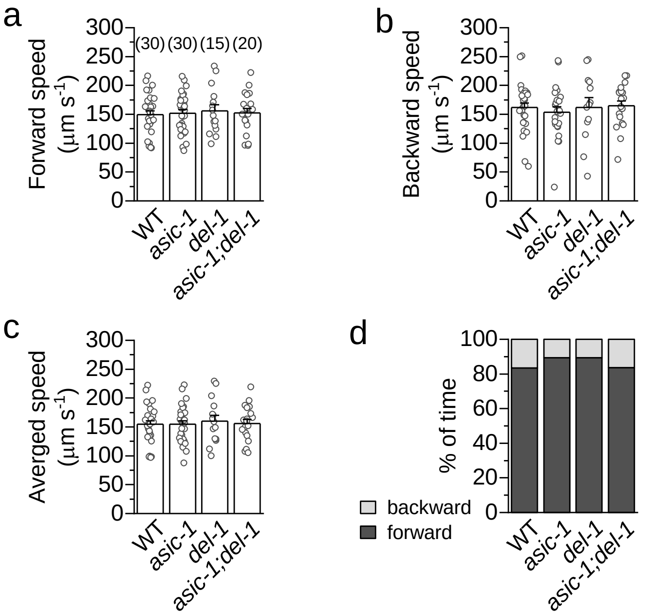

Supplementary Fig. 9. Mutations of *asic-1* and *del-1* do not alter locomotion behavior. Comparisons of forward speed (**a**), backward speed (**b**), averaged speed (**c**), and the percentage of the time spent on forward and backward movement (**d**) among wild type (WT), *asic-1(ok415)*, *del-1(ok150)*, and *asic-1(ok415);del-1(ok150)*. No statistically significant difference between WT and the mutants in all the comparisons (one-way ANOVA with Tukey's post hoc test). p = 0.9917, 0.937, and 0.99 (**a**), 0.8984, 1, and 0.9973 (**b**), 1, 0.9597, and 0.9993 (**c**), 0.4978, 0.6024, and 0.9885 (**d**). Brackets contain the number of animals tested. Data are shown as mean ± SEM. Source data are provided as a Source Data file.

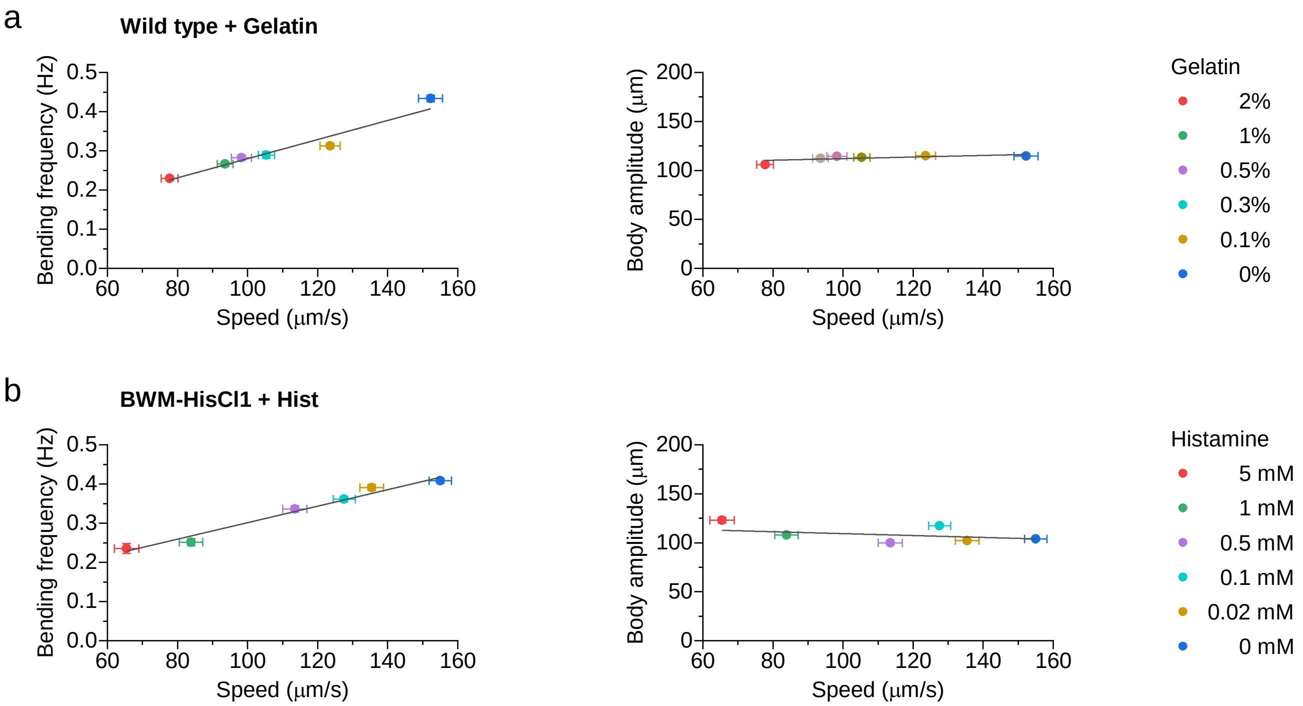

Supplementary Fig. 10. The bending frequency but not the body amplitude increases with the locomotion speed of *C. elegans*. a Relationships of locomotion speed versus bending frequency and body amplitude in wild type with gelatin added to assay plates at different concentrations. **b** Relationships of locomotion speed versus bending frequency and body amplitude in BWM-HistCl1 animals treated with different concentrations of histamine. Solid lines are linear fits to the data. Each animal was subjected to a single concentration of histamine or gelatin, and each data point represents results from 140-150 (**a**) or 96-112 (**b**) animals. Data are shown as mean ± SEM. Source data are provided as a Source Data file.

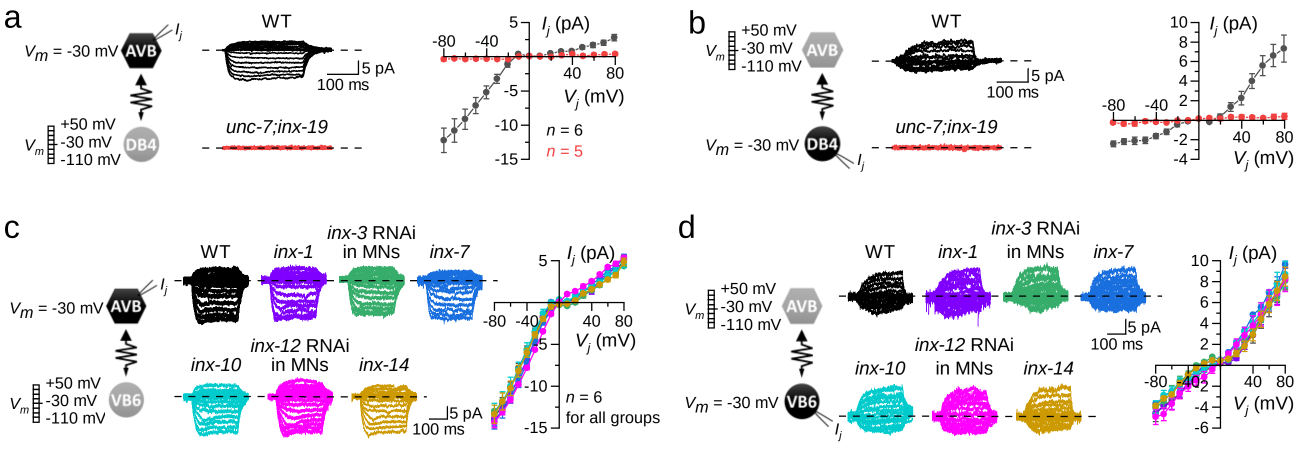

Supplementary Fig. 11. AVB and B-MN coupling is abolished in the *unc-7;inx-19* double mutant but normal in mutants of other candidate innexins. a, b Junctional currents (I_j) recorded from AVB (**a**) and DB4 (**b**) in response to membrane voltage steps applied to DB4 and AVB, respectively, in wild type (WT) and *unc-7(e5);inx-19(tm1896)* mutant. **c, d** I_j recorded from AVB (**c**) and VB6 (**d**) in response to membrane voltage steps applied to VB6 and AVB, respectively, in WT, *inx-1(tm3524)*, *inx-7(tm2738)*, *inx-10(ok2714)*, *inx-14(ag17)*, and two independent strains with motor neuron (MN)-targeted knockdown of *inx-3* and *inx-12*. Shown are sample I_j traces and transjunctional voltage (V_j) - I_j relationships. *n* represents the number of cell pairs recorded. Data are shown as mean ± SEM. Source data are provided as a Source Data file.

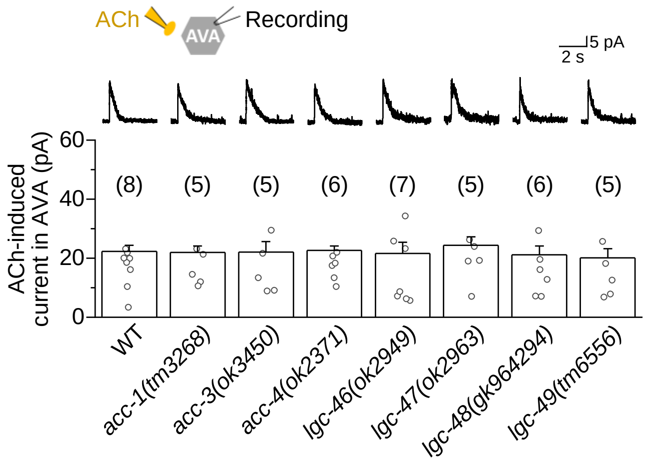

Supplementary Fig. 12. Acetylcholine (ACh)-induced outward current in AVA is normal in mutants of other candidate ACh-gated chloride channel genes. Shown are sample traces and statistical comparison among wild type (WT) and mutants. The whole-cell current was recorded at a holding potential of +30 mV using a low-Cl^- pipette solution (see Methods). No significant difference between WT and the mutants (one-way ANOVA with Tukey's post hoc test). p = 1, 1, 1, 1, 0.9994, 1, and 0.9994. Brackets contain the number of cells recorded. Data are shown as mean ± SEM. Source data are provided as a Source Data file.

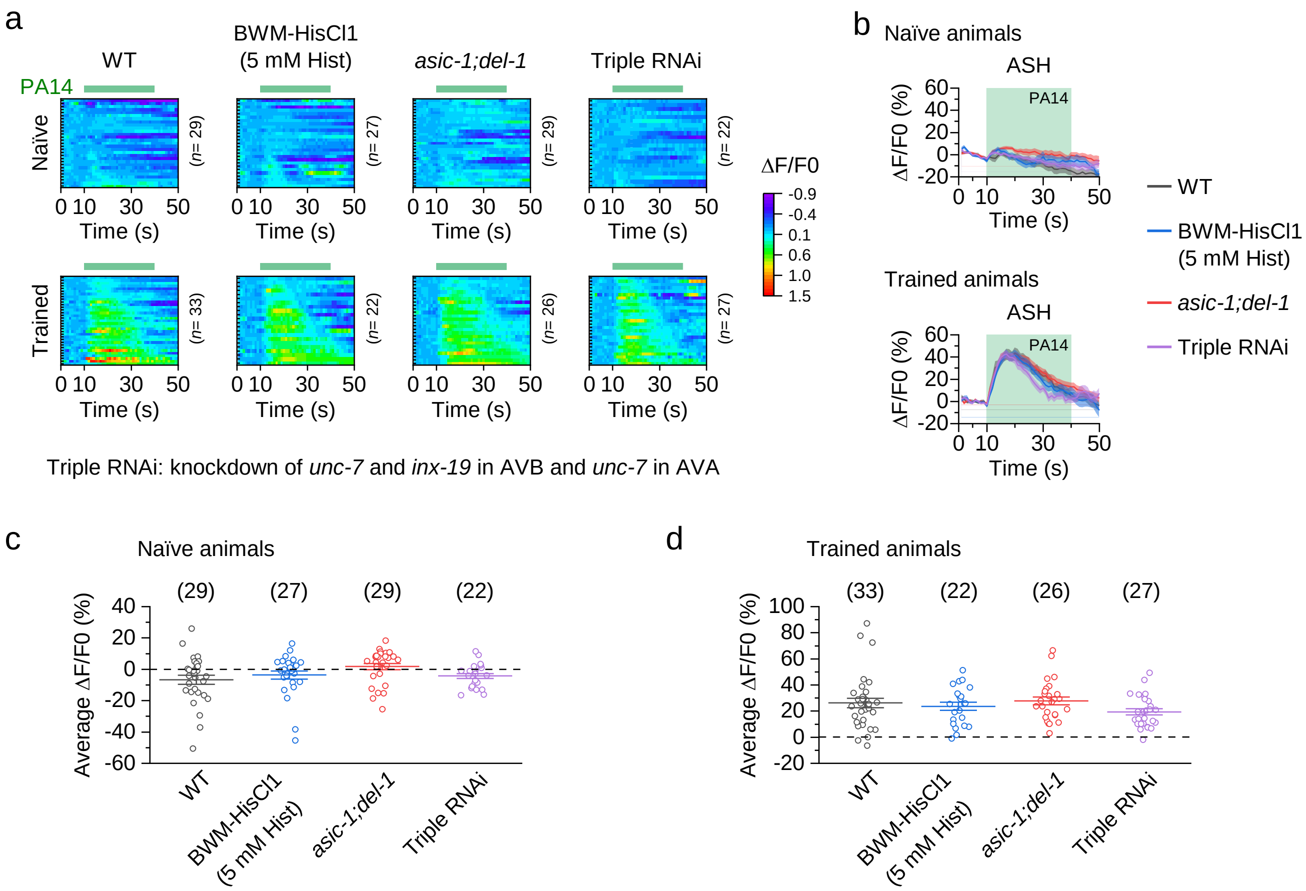

Supplementary Fig. 13. PA14-training-induced sensitization of ASH to PA14 stimulation is not affected by either locomotion inhibition, mechanoreceptor mutations, or disruption of gap junctions between locomotion interneurons and motor neurons. GCaMP6 signals in ASH were recorded from naïve and trained animals of wild type (WT), BWM-HisCl1 animals treated with histamine, *asic-1(ok415);del-1(ok150)*, and a triple knockdown strain (*unc-7* and *inx-19* in AVB, and *unc-7* in AVA). **a**. Heatmaps of Ca^{2+} signal (arranged by the response duration). Each row represents the ASH GCaMP6 signal of an individual animal. **b** The mean (solid line) and SEM (shaded area) of each group. **c, d** Comparison of average Ca^{2+} transient amplitude during 30-s of PA14 stimulation in naïve (**c**) and trained animals (**d**). No statistically significant difference among the four groups (one-way ANOVA with Tukey's post hoc test). Compared with WT, p = 0.7973, 0.0548, and 0.9085 (**c**), 0.9399, 0.9828, and 0.3902 (**d**). Brackets contain the number of ASH neurons analyzed (n). Data are shown as mean ± SEM. Source data are provided as a Source Data file.

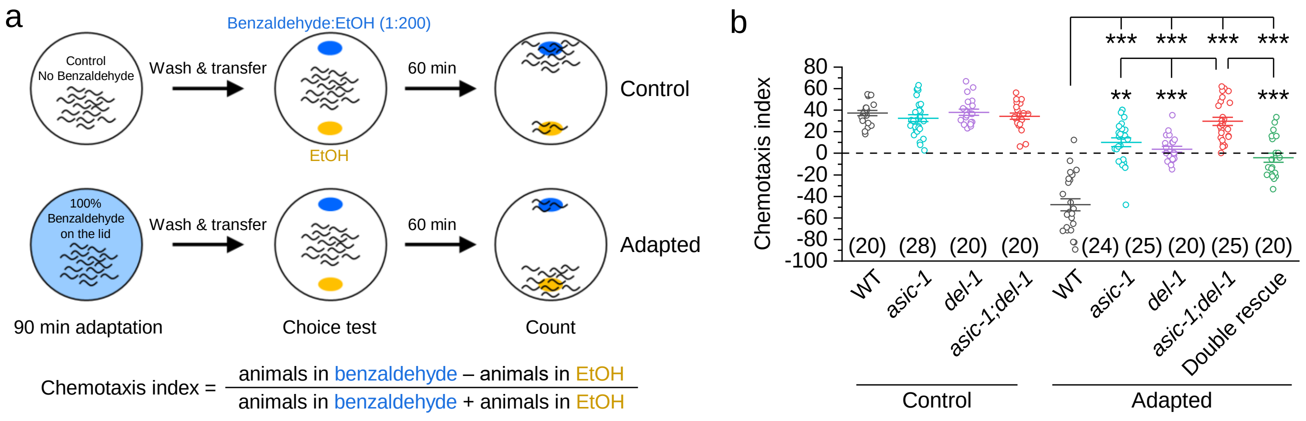

Supplementary Fig. 14. Mutations of mechanoreceptors impair olfactory adaptation to benzaldehyde. a Olfactory adaptation assay procedures shown schematically and the equation for calculating the chemotaxis index. **b** Comparison of the chemotaxis index among wild type (WT), *asic-1(ok415)*, *del-1(ok150)*, *asic-1(ok415);del-1(ok150)*, and the double mutant expressing wild-type *asic-1* and *del-1* in VA and VB under the control of *Pdel-1*. ** and *** indicate $p < 0.01$ and $p < 0.001$, respectively (one-way ANOVA with Tukey's post hoc test). In control group, p = 0.6341, 0.9988, and 0.9023 for comparison with WT. In adapted group, p = 0 for every comparison with WT, 0.0088 for *asic-1* versus *asic-1;del-1*, 0.0004 for *del-1* versus *asic-1;del-1*, < 0.0001 for *asic-1;del-1* versus double rescue. Brackets contain the number of independent assays. Data are shown as mean ± SEM. Source data are provided as a Source Data file.

Supplementary Table 1. Primers used in this study.

Name of DNA fragment	PCR product length	Sequence of primers (F, forward; R, reverse)
Pasic-1	3.7 kb	F: CAACTGCAGGTTGGAAAATCTAAAGATTTGC
		R: CTGGGTACCTTGGTGGCCTGAAATTTGATC
Pdel-1	2 kb	F: ATAGCATGCTCAAGTCCCACCTCAACCCCCG
		R: GTGACCGGTTCCAAAATTCCACATCTAG
Pdat-1	716 bp	F: CAACTGCAGAAGCTTCCATGAAATGGAACTTGAATCCAG
		R: ACAGGATCCGGCTAAAAATTGTTGAGATTCGAG
Pegl-46	3 kb	F: ACCAAGCTTAGTTCACGCCAGATGCAAGATG
		R: TTAGGATCCGGCCTTCTGAAATCAAAACG
Pser-2prom3	1.6 kb	F: ATTCTGCAGCGAAACGCTGTCGACTTCAAC
		R: CGCGGATCCGTTGTGATGTCACAAAAATATG
Psra-6	2.4 kb	F: TCTGAGGTGCATTTGCGA
		R: GGCAAAATCTGAAATAATAAATA
asic-1 (cDNA)	2472 bp	F: ATAACCGGTATGGGAAAGAACAGCTTAAAACGG
		R: TGTGCCGGCTCAATTATCAAGATTAAACCCG
del-1 (cDNA)	2085 bp	F: CATGGTACCATGCCATTCGGTGTCACAATGGG
		R: CGTGCCGGCTCAATTATTATTTGTGGATACTCC
inx-3 RNAi	450 bp	F: TCAGGATCCATGTTGGGTGTACCGTTCAT
		R: ATCGCCGGCCTCTGAGTCTTGAGACCGAA
inx-12 RNAi	450 bp	F: TCAACCGGTATGAACGTCATCCAGAATCT
		R: ATCGCCGGCTGTATTGCAAAGGGATGTCA
unc-7 RNAi	480 bp	F: TTAGGATCCACGTGAAATTTATTCGCGT
		R: AATGCCGGCCACTCAATCGCGTGCATC
unc-9 RNAi	415 bp	F: GTAACCGGTAGAAGCGCTACACGTGAAACATC
		R: AATGCTAGCGAAAAGCGAGCACAAAGTAGCAC
inx-19 RNAi	483 bp	F: TAAGGTACCATTAAGTGTCCGTGGAGATGATG
		R: TAAGCCGGCCCGTTCGGAGATTGTAGGTC
acc-2 (cDNA)	1338 bp	F: GTGACCGGTATGATATTTACTCTTTTATCAACACTGCC
		R: AATGCTAGCTTATCCGTCAACTCGATTGATCAAGTTCTG
acc-2 RANi	480 bp	F: GTGACCGGTTATTTACTCTTTTATCAACACTG
		R: AATGCTAGCTCGGGAAAAGCATCAAGAAA
lgc-55 (cDNA)	1745 bp	F: GTTGGTACCATGGTGTTCTCGTTCATTCTTAC
		R: ATGGCCGGCTTAATCCTTGGATTTTGCCGTAT
lgc-55 RANi	523 bp	F: CTCGGTACCTGGTGTTCTCGTTCATTCTTACT
		R: CTCGCCGGCACATTCGGTAGCCAGATATTATC
