# Supplementary material for: Locomotion modulates olfactory learning through proprioception in C. elegans
Source: Nat Commun. 2023 Jul 27;14:4534. doi: 10.1038/s41467-023-40286-x (PMC10374624; doi:10.1038/s41467-023-40286-x)
Supplement: Supplementary file 3 — Reporting Summary [file 41467_2023_40286_MOESM3_ESM.pdf]

nature portfolio

Corresponding author(s): Ping Liu

Last updated by author(s): Jul 8, 2023

Reporting Summary

Nature Portfolio wishes to improve the reproducibility of the work that we publish. This form provides structure for consistency and transparency in reporting. For further information on Nature Portfolio policies, see our Editorial Policies and the Editorial Policy Checklist.

Statistics

For all statistical analyses, confirm that the following items are present in the figure legend, table legend, main text, or Methods section.

n/a	Confirmed	
☐	☒	The exact sample size (*n*) for each experimental group/condition, given as a discrete number and unit of measurement
☐	☒	A statement on whether measurements were taken from distinct samples or whether the same sample was measured repeatedly
☐	☒	The statistical test(s) used AND whether they are one- or two-sided<br>*Only common tests should be described solely by name; describe more complex techniques in the Methods section.*
☒	☐	A description of all covariates tested
☒	☐	A description of any assumptions or corrections, such as tests of normality and adjustment for multiple comparisons
☐	☒	A full description of the statistical parameters including central tendency (e.g. means) or other basic estimates (e.g. regression coefficient) AND variation (e.g. standard deviation) or associated estimates of uncertainty (e.g. confidence intervals)
☐	☒	For null hypothesis testing, the test statistic (e.g. *F*, *t*, *r*) with confidence intervals, effect sizes, degrees of freedom and *P* value noted<br>*Give P values as exact values whenever suitable.*
☒	☐	For Bayesian analysis, information on the choice of priors and Markov chain Monte Carlo settings
☒	☐	For hierarchical and complex designs, identification of the appropriate level for tests and full reporting of outcomes
☒	☐	Estimates of effect sizes (e.g. Cohen's *d*, Pearson's *r*), indicating how they were calculated

Our web collection on statistics for biologists contains articles on many of the points above.

Software and code

Policy information about availability of computer code

Data collection	Clampex software (version 10, Molecular Devices) was used for electrophysiological recordings; NIS-Elements imaging software (version 4.51, Nikon) was used to control the on-and-off of light for optogenetic stimulation; MShot Image Analysis System (version 1.1, Mshot) was used for imaging collection; Track-A-Worm was used for locomotion tests and was previously published in Wang, S. J. and Wang, Z. W., 2013.
Data analysis	Electrophysiological data were quantified using Clampfit (version 10, Molecular Devices); Calcium imaging data were analyzed using ImageJ (version 1.50i, National Institutes of Health); The photobleaching-induced drop of the fluorescence signal was corrected using a custom MATLAB (version R2023a, MathWorks) module, which is freely available at https://health.uconn.edu/worm-lab/track-a-worm/; Locomotion behavior was analyzed using Track-A-Worm; Statistical analyses and data graphing were performed using Origin (version 2019, OriginLab).

For manuscripts utilizing custom algorithms or software that are central to the research but not yet described in published literature, software must be made available to editors and reviewers. We strongly encourage code deposition in a community repository (e.g. GitHub). See the Nature Portfolio guidelines for submitting code & software for further information.

Data

Policy information about availability of data

All manuscripts must include a data availability statement. This statement should provide the following information, where applicable:

- Accession codes, unique identifiers, or web links for publicly available datasets
- A description of any restrictions on data availability
- For clinical datasets or third party data, please ensure that the statement adheres to our policy

All data generated or analyzed during this study are included in this published article and its supplementary information files. Associated raw data are provided as Source data files of the main and supplementary figures.

Research involving human participants, their data, or biological material

Policy information about studies with human participants or human data. See also policy information about sex, gender (identity/presentation), and sexual orientation and race, ethnicity and racism.

Reporting on sex and gender	not applicable
Reporting on race, ethnicity, or other socially relevant groupings	not applicable
Population characteristics	not applicable
Recruitment	not applicable
Ethics oversight	not applicable

Note that full information on the approval of the study protocol must also be provided in the manuscript.

Field-specific reporting

Please select the one below that is the best fit for your research. If you are not sure, read the appropriate sections before making your selection.

☒ Life sciences ☐ Behavioural & social sciences ☐ Ecological, evolutionary & environmental sciences

For a reference copy of the document with all sections, see nature.com/documents/nr-reporting-summary-flat.pdf

Life sciences study design

All studies must disclose on these points even when the disclosure is negative.

Sample size	Sample sizes for electrophysiology were determined based on our previous experience (see, for example, Liu, P. et al., 2017; Liu, P. et al., 2020; Shui, Y. et al., 2020). Sample sizes for calcium imaging and behavioral assays were determined based on previous studies published in Jin, X. et al., 2016 and Choi, M. K. et al., 2020.
Data exclusions	No data were excluded from the analyses.
Replication	Replications were done by using different cells, different animals, and independent assays. All experiments were performed on at least two days. The n numbers for electrophysiology are the number of cells recorded. The n numbers for calcium imaging and locomotion tests are the number of animals tested; The n numbers for learning assays are the number of independent assays. All attempts at replication were successful.
Randomization	For each genotype, animals grown on the same plate were randomly allocated to different conditions. For individual assays, animals were randomly selected from the plates where they were grown or trained. For population assays, animals from a plate were washed off with M9 buffer, and adequate amount of buffer containing 100-150 animals was placed onto each choice plate.
Blinding	No.<br>All electrophysiological recordings were performed by two researchers. Blinding was not practical.<br>Locomotion tests were performed using an automated Track-A-Worm system. The role of the researcher is minimal.<br>Most animals used for calcium imaging and learning assays were easily distinguishable based on locomotion speed. Blinding was not applicable.

Reporting for specific materials, systems and methods

We require information from authors about some types of materials, experimental systems and methods used in many studies. Here, indicate whether each material, system or method listed is relevant to your study. If you are not sure if a list item applies to your research, read the appropriate section before selecting a response.

Materials & experimental systems

n/a	Involved in the study
☒	☐ Antibodies
☒	☐ Eukaryotic cell lines
☒	☐ Palaeontology and archaeology
☐	☒ Animals and other organisms
☒	☐ Clinical data
☒	☐ Dual use research of concern
☒	☐ Plants

Methods

n/a	Involved in the study
☒	☐ ChIP-seq
☒	☐ Flow cytometry
☒	☐ MRI-based neuroimaging

Animals and other research organisms

Policy information about studies involving animals; ARRIVE guidelines recommended for reporting animal research, and Sex and Gender in Research

Laboratory animals	All experiments were performed with young adult C. elegans hermaphrodites.The following C. elegans strains were used: N2 Wild-type Bristol, RB680 asic-1(ok415), NC279 del-1(ok150), RB1192 acd-2(ok1237), VC1047 acd-3(ok1335), RB1351 acd-4(ok1508), RB2005 acd-5(ok2657), RB557 asic-2(ok289), TU38 deg-1(u38), VC2633 degt-1(ok3307), RB1979 del-3(ok2613), RB1064 del-4(ok1014), RB1156 del-7(ok1187), VC831 del-8(ok1357), RB1818 del-9(ok2353), RB1469 del-10(ok1705), RB1177 delm-1(ok1226), RB1523 delm-2(ok1822), RB2521 egas-1(ok3497), VC975 egas-2(ok1477), RB1356 egas-3(ok1522), JC55 flr-1(ut11), TU253 mec-4(u253), ZB2551 mec-10(tm1552), ZW1013 unc-8(tm2071), RB1316 unc-105(ok1432), PLX214 asic-1(ok415);del-1(ok150), PLX425 asic-1(ok415);xyhEx425[Punc-17Δ1::asic-1(cDNA), Pmyo-2::mStrawberry], PLX270 del-1(ok150);xyhEX270[Punc-17Δ1::del-1(cDNA)::SL2::GFP, Pmyo-2::mStrawberry], PLX151 asic-1(ok415);del-1(ok150);xyhEx151[Punc-17Δ1::asic-1(cDNA)::SL2::mStrawberry, Punc-17Δ1::del-1(cDNA)::SL2::mStrawberry, Pmyo-2::GFP], PLX308 xyhIs308[Pmyo-3::HisCl1::SL2::GFP, Pmyo-2::mStrawberry], PLX39 xyhEx39[Ptdc-1::GCaMP6s, Pnpr-9::GCaMP6s, Pflp-18::loxP::LacZ::STOP::loxP::GCaMP6s, Pgpa-14::Cre, lin-15(+)], PLX228 xyhEx228[Pmyo-3::HisCl1::mStrawberry; Punc-122::dsRed];xyhEx39[Ptdc-1::GCaMP6s, Pnpr-9::GCaMP6s, Pflp-18::loxP::LacZ::STOP::loxP::GCaMP6s, Pgpa-14::Cre, lin-15(+)], PLX192 xyhEx192[Pasic-1::GFP, Pdel-1::SL2::mStrawberry], PLX238 xyhEx238[Pser-2prom3::GFP, Pasic-1::mStrawberry], PLX239 xyhEx239[Pdat-1::mStrawberry, Pasic-1::GFP], PLX240 xyhEx240[Pegl-46::mStrawberry, Pasic-1::GFP], PLX197 asic-1(ok415);del-1(ok150);xyhEx39[Ptdc-1::GCaMP6s, Pnpr-9::GCaMP6s, Pflp-18::loxP::LacZ::STOP::loxP::GCaMP6s, Pgpa-14::Cre, lin-15(+)], PLX303 asic-1(ok415);del-1(ok150);xyhEx39[Ptdc-1::GCaMP6s, Pnpr-9::GCaMP6s, Pflp-18::loxP::LacZ::STOP::loxP::GCaMP6s, Pgpa-14::Cre, lin-15(+)];xyhEx303[Pdel-1::SL2::asic-1(cDNA):: SL2::mStrawberry, Pdel-1::SL2::del-1(cDNA), Punc-122::dsRed], PLX306 asic-1(ok415);del-1(ok150);xyhEx306[Pdel-1::SL2::del-1(cDNA), Pdel-1::SL2::asic-1(cDNA)::SL2::mStrawberry, Pmyo-2::GFP], PLX310 asic-1(ok415);del-1(ok150);xyhIs308[Pmyo-3::HisCl1::SL2::GFP, Pmyo-2::mStrawberry], PLX24 xyhIs24[Psra-11::GFP], ZW230 inx-1(tm3524);zwIs142[Psra-11::GFP], ZW1452 zwEx286[Punc-17Δ1::: inx-3ss, Punc-17Δ1:: inx-3as];zwIs142[Psra-11::GFP(wp712)], ZW1314 inx-7(tm2738);zwIs142[Psra-11::GFP], ZW231 inx-10(ok2714);zwIs142[Psra-11::GFP], ZW1458 zwEx287[Punc-17Δ1::: inx-12ss, Punc-17Δ1:: inx-12as];zwIs142[Psra-11::GFP], ZW1315 inx-14(ag17);zwIs142[Psra-11::GFP], PLX273 unc-7(e5);xyhIs24[Psra-11::GFP], PLX274 unc-9(fc16);xyhIs24[Psra-11::GFP], PLX126 inx-19(tm1896);xyhEx42[Psra-11::GFP], PLX42 unc-7(e5);inx-19(tm1896);xyhEx42[Psra-11::GFP], PLX112 xyhEx112[Psra-11::unc-7ss, Psra-11::unc-7as, Psra-11::GFP], PLX370 xyhEx370[Pacr-5::unc-9ss, Pacr-5::unc-9as, Psra-11::GFP], PLX155 xyhEx155[Psra-11::inx-19ss, Psra-11::inx-19as, Psra-11::GFP], PLX118 xyhEx118[Pacr-11::unc-7ss, Pacr-11::unc-7as, Pasr-11::inx-19ss, Pasr-11::inx-19as, Pmyo-2::GFP], PLX373 xyhEx373[Psra-11::unc-7ss, Psra-11::unc-7as, Psra-11::inx-19ss, Psra-11::inx-19as, Psra-11::GFP], ZW704 zwEx175[Pflp-18::loxP::LacZ::STOP::loxP::mCherry::SL2::GFP, Pgpa-14::Cre], ZW731 unc-7(e5);zwEx175[Pflp-18::loxP::LacZ::STOP::loxP::mCherry::SL2::GFP, Pgpa-14::Cre], PLX203 xyhEx203[Pflp-18::loxP::LacZ::STOP::loxP::unc-7ss, Pflp-18::loxP::LacZ::STOP::loxP::unc-7as, Pgpa-14::Cre, Pmyo-2::GFP], ZW933 zwEX202[Pflp-18::loxP::LacZ::STOP::loxP::unc-7ss, Pflp-18::loxP::LacZ::STOP::loxP::unc-7as, Pgpa-14::Cre, Pmyo-2:: mStrawerry];zwEx175[Pflp-18::loxP::LacZ::STOP::loxP::mCherry::SL2::GFP, Pgpa-14::Cre], PLX449 xyhEx39[Ptdc-1::GCaMP6s, Pnpr-9::GCaMP6s, Pflp-18::loxP::LacZ::STOP::loxP::GCaMP6s, Pgpa-14::Cre, lin-15(+)];xyhEx449[Psra-11::unc-7ss, Psra-11::unc-7as, Psra-11::inx-19ss, Psra-11::inx-19as, Pflp-18::loxP::LacZ::STOP::loxP::unc-7ss, Pflp-18::loxP::LacZ::STOP::loxP::unc-7as, Pgpa-14::Cre, Punc-122::dsRed], PLX454 xyhIs307[Pmyo-3::HisCl1::SL2::GFP, Pmyo-2::mStrawberry];xyhEx454[Psra-11::unc-7ss, Psra-11::unc-7as, Psra-11::inx-19ss, Psra-11::inx-19as, Pflp-18::loxP::LacZ::STOP::loxP::unc-7ss, Pflp-18::loxP::LacZ::STOP::loxP::unc-7as, Pgpa-14::Cre, Pmyo-2::GFP], PLX200 xyhEx200[Psra-11::HisCl1::SL2::mStrawberry, Punc-122::dsRed];xyhEx39[Ptdc-1::GCaMP6s, Pnpr-9::GCaMP6s, Pflp-18::loxP::LacZ::STOP::loxP::GCaMP6s, Pgpa-14::Cre, lin-15(+)], PLX281 xyhIs281[Psra-11::GCaMP6s, Pflp-18::loxP::LacZ::STOP::loxP::GCaMP6s, Pgpa-14::Cre], ZW1191 kyEx3801[Psra-11::ChR2::GFP, Punc-122::dsRed];zwIs143[Pflp-18::loxP::LacZ::STOP::loxP::mStrawberry, Pgpa-14::Cre, lin-15(+)], XL238 ntIs[Prig-3::ChR2, Punc-122::dsRed];ntIs35[Psra-11::tdTomato];lite-1(ce314), ZW1185 acc-1(tm3268);zwEx175[Pflp-18::loxP::LacZ::STOP::loxP::mCherry::SL2::GFP, Pgpa-14::Cre], ZW1186 acc-2(ok2216);zwEx175[Pflp-18::loxP::LacZ::STOP::loxP::mCherry::SL2::GFP, Pgpa-14::Cre], ZW1183 acc-3(ok3450);zwEx175[Pflp-18::loxP::LacZ::STOP::loxP::mCherry::SL2::GFP, Pgpa-14::Cre], ZW1184 acc-4(ok2371);zwEx175[Pflp-18::loxP::LacZ::STOP::loxP::mCherry::SL2::GFP, Pgpa-14::Cre], ZW1215 lgc-46(ok2949);zwEx175[Pflp-18::loxP::LacZ::STOP::loxP::mCherry::SL2::GFP, Pgpa-14::Cre], ZW1220 lgc-47(ok2963);zwEx175[Pflp-18::loxP::LacZ::STOP::loxP::mCherry::SL2::GFP, Pgpa-14::Cre], ZW1229 lgc-48(gk964294);zwEx175[Pflp-18::loxP::LacZ::STOP::loxP::mCherry::SL2::GFP, Pgpa-14::Cre], ZW1181 lgc-49(tm6556);zwEx175[Pflp-18::loxP::LacZ::STOP::loxP::mCherry::SL2::GFP, Pgpa-14::Cre], ZW1196 acc-2(ok2216);kyEx3801[Psra-11::ChR2::GFP, Punc-122::dsRed];zwIs143[Pflp-18::loxP::LacZ::STOP::loxP::mStrawberry, Pgpa-14::Cre,

lin-15(+)], ZW1450 acc-2(ok2216);kyEx3801[Psra-11::ChR2::GFP, Punc-122::dsRed];zwEx277[Pflp-18::loxP::LacZ::STOP::loxP::acc-2(cDNA), Pflp-18::loxP::LacZ::STOP::loxP::mCherry::SL2::GFP, Pgpa-14::Cre], ZW1469 kyEx3801[Psra-11::ChR2::GFP, Punc-122::dsRed];zwEx284[Pflp-18::loxP::LacZ::STOP::loxP::acc-2 ss;Pflp-18::loxP::LacZ::STOP::loxP::acc-2 as;Pflp-18::loxP::LacZ::STOP::loxP::mCherry::SL2::GFP, Pgpa-14::Cre], PLX264 xyhEx264[Pflp-18::loxP::LacZ::STOP::loxP::acc-2ss, Pflp-18::loxP::LacZ::STOP::loxP::acc-2as, Pgpa-14::Cre, Pmyo-2::GFP], PLX285 xyhIs281[Psra-11::GCaMP6s, Pflp-18::loxP::LacZ::STOP::loxP::GCaMP6s, Pgpa-14::Cre, Punc-122::deRed];xyhEx285[Pflp-18::loxP::LacZ::STOP::loxP::acc-2ss, Pflp-18::loxP::LacZ::STOP::loxP::acc-2as, Punc-122::dsRed], PLX84 lgc-55(n4331);xyhIs24[Psra-11::GFP], PLX275 lgc-55(n4331);xyhEx275[Psra-11::lgc-55(cDNA), Psra-11::GFP, Punc-122::dsRed], PLX278 xyhEx278[Psra-11::lgc-55ss, Psra-11::lgc-55as, Psra-11::GFP, Pmyo-2::GFP], PLX267 xyhEx267[Psra-11::lgc-55ss, Psra-11::lgc-55as, Pmyo-2::GFP], PLX288 xyhIs281[Psra-11::GCaMP6s, Pflp-18::loxP::LacZ::STOP::loxP::GCaMP6s, Pgpa-14::Cre];xyhEx288[Psra-11::lgc-55ss, Psra-11::lgc-55as, Punc-122::dsRed], PLX348 xyhEx348[Psra-6::GCaMP6s, Punc-122::dsRed], PLX455 asic-1(ok415);del-1(ok150);xyhEx348[Psra-6(2.4kb)::GCaMP6s, Punc-122::dsRed], PLX456 xyhEx456[Pmyo-3::HisCl1::SL2::mStrawberry, Psra-6::GCaMP6s, Punc-122::GFP], PLX459 xyhEx348[Psra-6::GCaMP6s, Punc-122::dsRed];xyhEx459[Psra-11::unc-7ss, Psra-11::unc-7as, Psra-11::inx-19ss, Psra-11::inx-19as, Pflp-18::loxP::LacZ::STOP::loxP::unc-7ss, Pflp-18::loxP::LacZ::STOP::loxP::unc-7as, Pgpa-14::Cre, Punc-122::GFP]

Wild animals	The study did not involve wild animals.
Reporting on sex	All experiments were performed with young adult hermaphrodites.
Field-collected samples	The study did not involve samples collected from the field.
Ethics oversight	The study uses the nematode C. elegans, which does not require ethical approval or guidance.

Note that full information on the approval of the study protocol must also be provided in the manuscript.
